# Interpretable machine learning analysis of immune-inflammatory biomarkers for predicting no-reflow in patients with acute myocardial infarction after percutaneous coronary intervention

**DOI:** 10.1080/07853890.2026.2747562

**Published:** 2026-10-16

**Authors:** Rensong Liu, Yan Peng, Haoran Qin, Yang Zhou, Xiaolong Li, Hongqian Wang, Ying Li, Hanlin Zhang, Xinyi Hu, Zhouqing Zhang, Weiran Ren, Xianlong Shu, Junhao Zhang, Li Nie, Xinliang Zou, Tao Jing

**Affiliations:** a Department of Cardiovascular Medicine, Southwest Hospital, Army Medical University, Chongqing, China; b Key Laboratory of Geriatric Cardiovascular and Cerebrovascular Disease, Ministry of Education of China, Chongqing, China; c Key Laboratory of Chronobiology and Cardiometabolic Disease, Chongqing Education Commission of China, Chongqing, China; d Center for Medical Big Data and Artificial Intelligence, Southwest Hospital, Army Medical University, Chongqing, China; e Medical Team, Xiangyang Detachment of the Armed Police Force, Hubei, China; f School of Medicine, Chongqing University, Chongqing, China

**Keywords:** Acute myocardial infarction, percutaneous coronary intervention, no-reflow, immune-inflammatory biomarkers, interpretable machine learning

## Abstract

**Objective:**

To evaluate whether routine immune-inflammatory biomarkers predict no-reflow (NR) after percutaneous coronary intervention (PCI) in acute myocardial infarction (AMI) patients, quantify their incremental predictive value over clinical variables, and build an interpretable peri-procedural prediction model.

**Methods:**

This prospective cohort recruited 382 AMI patients undergoing PCI. Univariable and multivariable logistic regression identified independent correlates; confounder-adjusted restricted cubic splines (RCS) assessed nonlinear associations. LASSO regression selected core predictors with variance inflation factor (VIF) assessing multicollinearity. Four machine-learning models underwent 1000-iteration bootstrap internal validation and SHAP interpretation. Incremental value was tested via ΔAUC (DeLong test), continuous NRI, and IDI. Stratified regression and interaction tests evaluated AMI-subtype effect modification.

**Results:**

NR occurred in 91 patients (23.8%). Multiple neutrophil-centred indices independently correlated with NR after adjustment. Seven predictors constituted the final model (all VIF < 2.5). LightGBM yielded optimal discrimination and modest net clinical benefit (AUC 0.799, 95% CI 0.699–0.899); AMI subtype and neutrophil-related markers dominated SHAP outputs. Neutrophil count, NAR, NHR, SII, and PIV significantly improved discrimination. Seven of the 16 tested markers yielded significant continuous NRI, and ten demonstrated significant IDI gains. In stratified analyses, neutrophil count, NLR, and MHR were associated with NR in STEMI but not NSTEMI patients; interaction tests found no significant AMI-subtype effect modification.

**Conclusion:**

Pre-procedural neutrophil-centred inflammatory biomarkers are independent correlates of post-PCI NR and offer incremental predictive value over conventional clinical variables. The interpretable LightGBM model supports peri-procedural NR risk stratification, though external validation and recalibration are mandatory before clinical application.

## Introduction

Acute myocardial infarction (AMI) remains a leading cause of global cardiovascular mortality and morbidity, with primary percutaneous coronary intervention (PCI) as the standard reperfusion strategy. Despite successful epicardial recanalisation of infarct-related arteries (IRA), microcirculatory perfusion impairment frequently leads to post-percutaneous coronary intervention no-reflow (NR) [1]. Beyond a transient angiographic abnormality, NR causes persistent microvascular injury, increases infarct size, and deteriorates long-term prognosis [2,3]. Even with contemporary stent techniques and potent antiplatelet therapy, NR strongly predicts adverse clinical outcomes, underscoring the need for accurate and clinically applicable risk prediction approaches [4]. Inflammation is pivotal to NR pathogenesis, wherein neutrophil-driven systemic inflammation predominantly mediates microcirculatory damage [5]. Routine blood-based composite inflammatory indices are cost-effective and readily accessible biomarkers for evaluating inflammatory burden; however, most previous studies have focused on limited individual markers and lack systematic head-to-head comparisons of multiple composite inflammatory indices [6–8].

Furthermore, existing NR prediction models commonly adopt uninterpretable machine learning frameworks, restricting their practical bedside implementation [9]. Accordingly, this prospective cohort study systematically compared the predictive performance of 15 composite immune-inflammatory indices and neutrophil count for post-PCI NR. We further quantified their incremental predictive value over conventional clinical variables and constructed an interpretable machine learning model to optimise peri-procedural NR risk stratification in AMI patients undergoing PCI.

## Methods

### Study design

This was a single-centre, prospective cohort study. Consecutive patients with AMI undergoing PCI at the First Affiliated Hospital (Southwest Hospital) of Army Medical University from January 1, 2023, to March 31, 2024, were enrolled if they met the inclusion criteria. The study was approved by the Ethics Committee of the First Affiliated Hospital of Army Medical University, Chongqing, China (Approval No. AKY2022111). Written informed consent was obtained from all participants, and the study was conducted in accordance with the Declaration of Helsinki.

### Inclusion criteria

1. Diagnosis of AMI was confirmed by clinical symptoms, electrocardiogram (ECG) findings, myocardial biomarker levels, and coronary angiography, and PCI was performed in accordance with the 2023 European Society of Cardiology (ESC) guidelines [10];
2. Age ≥ 18 years;
3. Coronary angiography showed at least one IRA with stenosis ≥ 50%;
4. No contraindications to PCI and successful completion of IRA recanalisation by PCI.

### Exclusion criteria

1. Age < 18 years;
2. Failure to achieve myocardial revascularisation during the procedure;
3. Poor-quality coronary angiography images that prevent accurate perfusion assessment;
4. Estimated glomerular filtration rate (eGFR) < 30 mL/min/1.73 m^2^;
5. Comorbid with autoimmune diseases, malignant tumours, severe infections, or haematologic disorders;
6. Recent use of anti-inflammatory drugs or immunosuppressants (within 2 weeks before enrolment);
7. Missing key clinical or laboratory data.

###  Definition of outcome events

The primary outcome was NR after PCI, which was defined as follows: after excluding mechanical obstruction factors (e.g. vascular dissection, vasospasm, thrombosis, or residual stenosis ≥50%), the Thrombolysis in Myocardial Infarction (TIMI) flow grade of the IRA was ≤2 [11]. The composite outcome was impaired myocardial perfusion, which was diagnosed if any of the following criteria were met: 1. Persistent or recurrent chest pain postoperatively (excluding in-stent thrombosis or vascular dissection) [12]; 2. ST-segment resolution (STR) <50% on ECG at 2 h postoperatively (excluding ventricular aneurysm) [13]; 3. pre-procedural IRA Index of Microcirculatory Resistance (IMR) <25 and post-procedural IMR ≥25 [14].

### Clinical data collection and perioperative management

All PCI procedures and perioperative management followed current ESC guidelines [10] and were performed by interventional cardiologists with at least 5 years of experience. IRA perfusion was assessed by coronary angiography immediately after PCI. TIMI flow grades were independently evaluated by two senior physicians, with a third senior physician adjudicating any disagreement.

**Data collection comprised three parts:** 1. Baseline clinical characteristics: sex, age (years), height (m), weight (kg), history of hypertension (HTN), history of diabetes mellitus (DM), and smoking history; 2. Pre-procedural laboratory parameters: platelet count (PLT,10^9^/L), neutrophil count (Neut,10^9^/L), lymphocyte count (Lymph, 10^9^/L), monocyte count (Mono, 10^9^/L), albumin (ALB, g/L), high-sensitivity C-reactive protein (hs-CRP, mg/L), uric acid (UA, μmol/L), high-density lipoprotein cholesterol (HDL-C, mmol/L), and low-density lipoprotein cholesterol (LDL-C, mmol/L); 3. Procedural and angiographic-related data: AMI subtype, SYNTAX score, pre-TIMI grade, and number of stents. All laboratory results were obtained from routine clinical tests at enrolment. Assays were performed using commercial reagents (Sysmex, Japan; Beckman Coulter, USA) on corresponding automatic biochemical analysers, with intra-assay and inter-assay coefficients of variation (CV) below 5%, and strict internal quality control was conducted in accordance with the Clinical and Laboratory Standards Institute (CLSI) guidelines.

### Calculation of derived indicators

Body mass index (BMI) and 15 composite immune-inflammatory indices were calculated based on the above laboratory parameters, and all formulas were derived from previously published studies as follows:

$$\text{BMI}=\text{ Weight}/{\text{Height}}^{2}$$

Systemic Immune−Inflammation Index (SII)=(PLT × Neut)/Lymph

Systemic Inflammatory Response Index (SIRI)=(Neut × Mono)/Lymph

Neutrophil−to−Lymphocyte Ratio (NLR)= Neut/Lymph

Platelet−to−Lymphocyte Ratio (PLR)= PLT/Lymph

Platelet−to−Neutrophil Ratio (PNR)= PLT/Neut

Neutrophil−to−HDL−C Ratio (NHR)= Neut/HDL−C

Monocyte−to−HDL−C Ratio (MHR)= Mono/HDL−C

Platelet−to−HDL−C Ratio (PHR)= PLT/HDL−C

Lymphocyte−to−HDL−C Ratio (LHR)= Lymph/HDL−C

Neutrophil−to−Monocyte Ratio (NMR)= Neut/Mono

CRP−to−Albumin Ratio (CAR)=hs−CRP/ALB

Uric Acid−to−Albumin Ratio (UAR)= UA/ALB

Lymphocyte−to−Albumin Ratio (LAR)= Lymph/ALB

Neutrophil−to−Albumin Ratio (NAR)= Neut/ALB

Pan−Immune−Inflammation Value (PIV)=(Neut × PLT × Mono)/Lymph

### Statistical analysis

IBM SPSS 26.0, R 4.2.0 (RStudio), and Python 3.13.5 were used for data cleaning and statistical analysis. The significance level was set at *α* = 0.05, and a two-sided *p* < 0.05 was considered statistically significant. Variables with more than 30% missing values were excluded; the remaining missing data were imputed by multiple imputation (*m* = 5 imputation iterations) using the mice package with random forest algorithms in R. The outcome variable (post-PCI NR) was not imputed. The Shapiro-Wilk test was used to assess the normality of data distribution. Normally distributed continuous variables were expressed as mean ± standard deviation (mean ± SD), non-normally distributed continuous variables as median (interquartile range) [M (Q1, Q3)], and categorical variables as number (percentage) [*n* (%)]. The independent-samples *t*-test was used for comparing normally distributed continuous variables between groups, the Mann-Whitney *U* test for non-normally distributed continuous variables, and the *χ^2^* test or Fisher’s exact test for categorical variables.

Univariable and multivariable logistic regression analyses of routine laboratory indicators and inflammatory composite indices were performed to identify independent predictors of NR. All multivariable models were adjusted for age, sex, hypertension, diabetes mellitus, smoking status, body mass index, SYNTAX score, pre-procedural TIMI grade, number of implanted stents, and AMI subtype.

Restricted cubic spline (RCS) regression with four knots placed at the 5th, 35th, 65th, and 95th percentiles was applied to characterise dose-response relationships between continuous biomarkers and NR risk, with tests for overall association and nonlinearity.

### Variable selection and model development

L1-penalised least absolute shrinkage and selection operator (LASSO) regression was applied mainly to reduce the number of candidate predictors and mitigate multicollinearity for subsequent model construction.

The predictor pool included candidate laboratory biomarkers and clinical/procedural variables used for multivariable regression adjustment (age, sex, hypertension, diabetes, smoking, BMI, SYNTAX score, pre-procedural TIMI grade, stent number and AMI subtype). Stratified 5-fold cross-validation was adopted to determine the optimal regularisation parameter *λ*.

For all predictors retained in the LASSO regression, we further calculated the variance inflation factor (VIF), and variables with VIF ≥ 10 were excluded to eliminate severe multicollinearity. Upon completion of the entire predictor selection workflow, the events-per-variable (EPV) ratio was assessed prior to model construction, with an EPV threshold of ≥10 pre-defined as the minimum requirement for eligible prediction model development.

Four prediction models were constructed using the final fixed set of selected predictors: logistic regression (LR), random forest (RF), extreme gradient boosting (XGBoost), and Light Gradient Boosting Machine (LightGBM). Bayesian hyperparameter optimisation was performed using stratified 5-fold cross-validation across the study cohort. The objective function for optimisation was the out-of-fold (OOF) F1-score, for which the optimal classification threshold for F1 calculation was independently determined by Youden’s index derived from the receiver operating characteristic (ROC) curve within each cross-validation fold to avoid data leakage. For each model, hyperparameter optimisation comprised 8 initial random parameter samples followed by 20 iterative Bayesian optimisation cycles, yielding a total of 28 parameter evaluations. Optimised hyperparameters for all four models are provided in Table S1.

### Model evaluation and interpretation

Internal validation was performed using 1000 bootstrap resamples of the full cohort. In each iteration, each model with a fixed predictor set and pre-specified hyperparameters was refitted on the bootstrap sample and evaluated on out-of-sample observations. Because predictor selection and hyperparameter tuning were performed once on the full cohort and held constant across resamples, this procedure quantifies the uncertainty of model fitting but does not propagate the uncertainty arising from predictor selection or hyperparameter tuning.

Model performance was assessed across three domains: discrimination, calibration, and clinical utility. Discrimination was evaluated using the area under the receiver operating characteristic curve (AUC), alongside the precision-recall (PR) curve and PR-AUC as complementary metrics for the imbalanced NR outcome. Calibration was assessed using calibration curves, calibration intercept, calibration slope, and Brier score. Clinical net benefit was quantified *via* decision curve analysis (DCA). Additional metrics included accuracy, precision, sensitivity, specificity, F1-score, and geometric mean (G-mean). The 95% confidence intervals (CIs) for all performance metrics, including AUC, were derived from the 2.5th and 97.5th percentiles of the 1000 bootstrap resamples. The optimal model was selected by average ranking across the nine pre-specified performance metrics (AUC, PR-AUC, Brier score, accuracy, precision, sensitivity, specificity, F1 and G-mean).

SHapley Additive exPlanations (SHAP) analysis was applied to the selected optimal model to quantify the direction and magnitude of each predictor’s contribution to individual-level risk predictions. SHAP dependence plots were generated to visualise the association between each predictor value and its corresponding SHAP value. To facilitate high-risk stratification for NR, the optimal probability cut-off value was determined by maximising Youden’s index using predicted probabilities derived from the selected optimal model.

### Assessment of incremental predictive performance of candidate inflammatory biomarkers

To evaluate the incremental predictive value of immune-inflammatory biomarkers for post-PCI NR in patients with acute myocardial infarction (AMI), the 15 composite immune-inflammatory indices together with neutrophil count were incorporated into the baseline clinical prediction model to construct extended models. The increment in the area under the receiver operating characteristic curve (ΔAUC) was calculated to quantify the improvement in model discrimination, and the DeLong test was performed to compare AUC values between the baseline clinical model and the extended model. Continuous net reclassification improvement (NRI) and integrated discrimination improvement (IDI) were further computed to assess improvements in risk reclassification capacity and overall predictive performance, respectively. Corresponding 95% confidence intervals were reported for continuous NRI and IDI. All statistical analyses were two-tailed, and *p* < 0.05 was considered statistically significant. These incremental analyses were predefined as exploratory, with no adjustment for multiple testing.

### Subgroup and interaction analysis

Two analytical components were performed in this section: subgroup association analyses and interaction testing. Patients were stratified into STEMI and NSTEMI subgroups for exploratory subgroup analyses. Logistic regression was performed within each subgroup to calculate odds ratios (ORs) and 95% confidence intervals (95% CIs) describing the association between candidate predictors and post-PCI NR. Interaction between each predictor and AMI subtype (STEMI vs NSTEMI) was assessed in the overall cohort using likelihood-ratio tests comparing nested models with and without the corresponding interaction term to obtain *P*-interaction values. An uncorrected *P*‑interaction <0.05 was regarded as nominally statistically significant.

## Results

### Baseline characteristics

Among the 382 enrolled patients, 91 (23.8%) developed post-PCI NR and 291 (76.2%) exhibited normal coronary flow (Figure 1). No significant between-group differences were observed for age, sex, body mass index, smoking status, diabetes, lipid profile, uric acid, lymphocyte count, PLR, LHR, NMR, UAR, LAR, SYNTAX score, or stent number (all *p* > 0.05).

**Figure 1.**
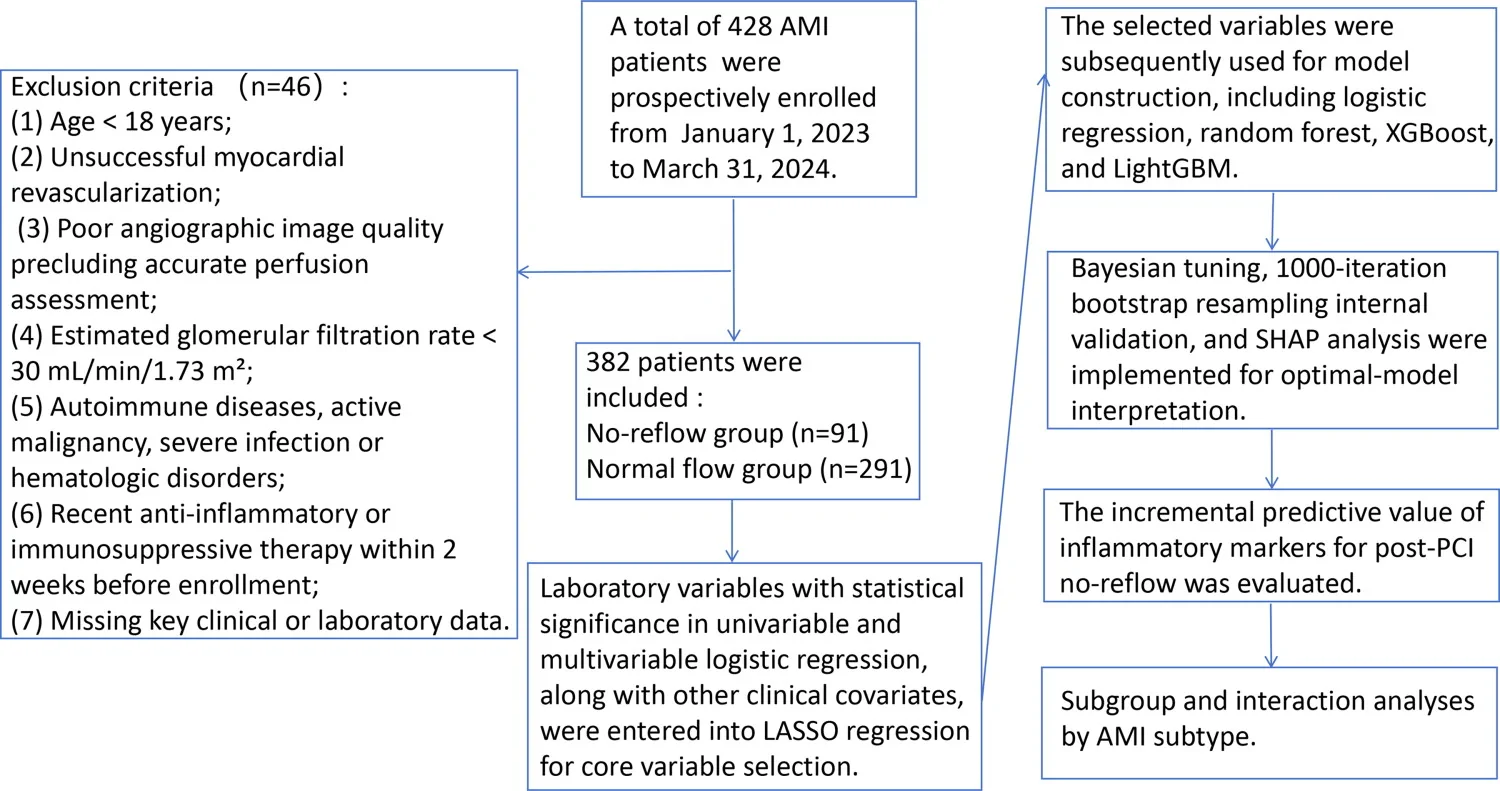
Flowchart of patient enrolment and the analytical pipeline. A total of 428 consecutive AMI patients were prospectively screened, of whom 46 were excluded according to the prespecified criteria, leaving 382 patients for the final analysis (no-reflow, *n* = 91; normal flow, *n* = 291).

Compared with the normal coronary flow group, the NR group had significantly higher levels of hs-CRP and albumin, and higher counts of monocytes, platelets and neutrophils. Multiple composite inflammatory indices, including SII, SIRI, NLR, NHR, MHR, PHR, CAR, NAR and PIV, were also elevated in the NR group, whereas PNR was lower (all *p* < 0.05). Patients in the NR group were more likely to present with STEMI (78.0% vs. 39.9%, *p* < 0.001) and had worse pre-procedural TIMI flow (*p* = 0.004) (Table S2).

### Univariable and multivariable logistic regression

Univariable analysis identified 16 laboratory variables significantly associated with NR. After multivariable adjustment, 12 variables including albumin, monocyte count, neutrophil count, SII, SIRI, NLR, PNR, NHR, MHR, PHR, NAR and PIV remained independent correlates of NR, whereas uric acid, hs-CRP, platelet count and CAR—significant in univariable analysis—lost independent significance. NAR showed a markedly elevated odds ratio (OR) with an extremely wide 95% CI (OR = 51.947, 95% CI: 3.860–699.039, *p* = 0.003), indicating substantial statistical instability of this effect estimate (Table 1).

**Table 1.** Univariable and multivariable logistic regression analyses for post-PCI NR.

Characteristics	Univariable analysis			Multivariable analysis		
	Odds Ratio	95% CI	*p*	Odds ratio	95% CI	*p*
LDL-C	0.925	(0.729 – 1.174)	0.521	–	–	–
UA	1.002	(1.000 – 1.004)	0.047*	1.002	(1.000–1.004)	0.073
ALB	1.077	(1.018 – 1.140)	0.011*	1.085	(1.021–1.154)	0.009*
hs-CRP	1.007	(1.003 – 1.011)	0.001*	1.005	(1.000–1.009)	0.057
HDL-C	0.692	(0.315 – 1.520)	0.359	–	–	–
Mono	3.639	(2.043 – 6.482)	<0.001*	2.726	(1.464–5.077)	0.002*
PLT	1.003	(1.001 – 1.006)	0.019*	1.003	(1.000–1.006)	0.063
Neut	1.173	(1.101 – 1.249)	<0.001*	1.136	(1.060–1.217)	<0.001*
Lymph	0.985	(0.916 – 1.060)	0.685	–	–	–
SII	1.000	(1.000 – 1.001)	<0.001*	1.000	(1.000–1.001)	0.005*
SIRI	1.142	(1.073 – 1.215)	<0.001*	1.115	(1.042–1.192)	0.002*
NLR	1.134	(1.055 – 1.220)	0.001*	1.124	(1.035–1.221)	0.005*
PLR	1.002	(0.999 – 1.005)	0.163	–	–	–
PNR	0.977	(0.963 – 0.992)	0.002*	0.982	(0.967–0.998)	0.026*
NHR	1.152	(1.084 – 1.225)	<0.001*	1.122	(1.049–1.201)	0.001*
MHR	3.189	(1.865 – 5.451)	<0.001*	2.585	(1.434–4.662)	0.002*
PHR	1.003	(1.001 – 1.005)	0.007*	1.003	(1.000–1.005)	0.049*
LHR	0.990	(0.935 – 1.048)	0.730	–	–	–
NMR	1.047	(0.995 – 1.102)	0.075	–	–	–
CAR	1.296	(1.097 – 1.531)	0.002*	1.198	(0.999–1.437)	0.052
UAR	1.031	(0.961 – 1.106)	0.390	–	–	–
LAR	0.318	(0.008 – 13.302)	0.548	–	–	–
NAR	150.966	(13.461 – 1693.110)	<0.001*	51.947	(3.860–699.039)	0.003*
PIV	1.000	(1.000 – 1.001)	0.001*	1.000	(1.000–1.001)	0.008*

*Note:* Univariable analysis was performed using unadjusted logistic regression. Multivariable logistic regression was adjusted for age, sex, hypertension, diabetes mellitus, smoking status, BMI, SYNTAX score, pre-procedural TIMI grade, stent number, and AMI subtype. Variables with *p* < 0.05 in univariable analysis were included in the multivariable model. OR, odds ratio; 95% CI, 95% confidence interval. OR values are presented to three decimal places. Statistical significance was defined as *p* < 0.05. The symbol “−” indicates variables excluded from multivariable analysis due to non-significant univariable results. *p < 0.05.

### Dose–response relationships

RCS analysis was applied to the 12 biomarkers that remained independently associated with NR in multivariable regression. Of these, all except PHR showed significant overall associations (all *P*-overall < 0.05; PHR: *P*-overall = 0.206). Statistically significant nonlinear relationships were observed for ALB (*P*-nonlinear = 0.018), SII (*P*-nonlinear = 0.024), and PIV (*P*-nonlinear = 0.010). NAR showed borderline nonlinearity (*P*-nonlinear = 0.050).

Albumin exhibited a *U*-shaped association, with both low and very high albumin levels corresponding to elevated NR risk and the risk nadir at intermediate albumin concentrations. SII displayed an initial steep rise followed by a plateau, while PIV showed a rapid initial increase followed by a slowly rising non-monotonic pattern. Monocyte count, neutrophil count, SIRI, NLR, NHR and MHR showed approximately monotonic positive dose-response associations. PNR had a significant overall effect without evident nonlinearity (*P*-overall = 0.047, *P*-nonlinear = 0.324) (Figure 2).

**Figure 2.**
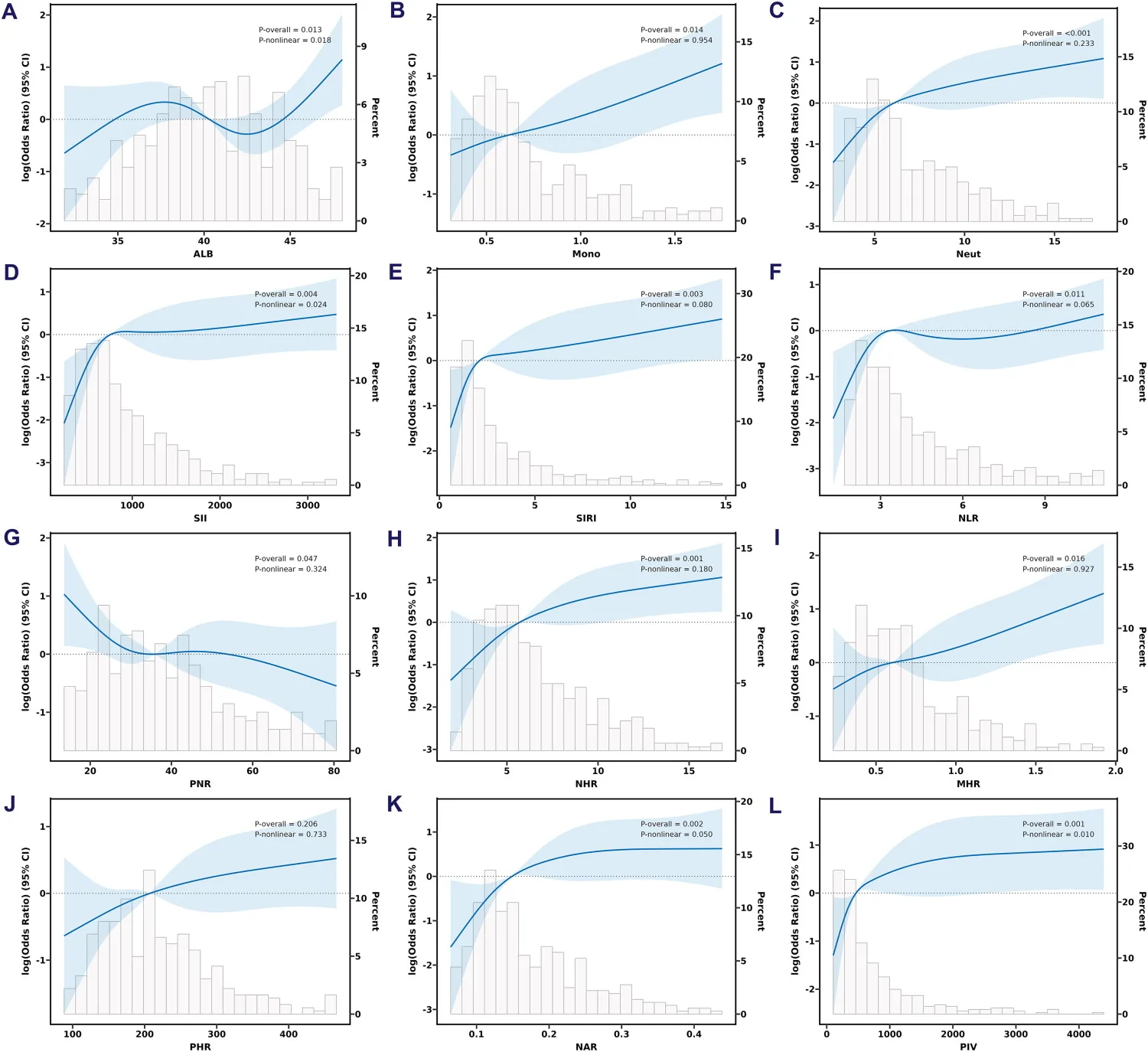
RCS analysis for the associations between nutritional and inflammatory markers and post-PCI NR risk. Odds ratios (solid lines) and corresponding 95% confidence intervals (shaded areas) were adjusted for age, sex, hypertension, diabetes mellitus, smoking status, BMI, SYNTAX score, pre-TIMI grade, number of stents, and AMI subtype. The horizontal dashed line represents OR = 1. Histograms illustrate the distribution of each marker within the study cohort. *P*-overall and *P*-nonlinear values are presented in each panel.

### Variable selection and multicollinearity

At the optimal *λ*.min = 0.0258, seven variables with non-zero coefficients were retained by LASSO regression: albumin, neutrophil count, NLR, MHR, hypertension, stent number, and AMI subtype. Given 91 NR events and these seven final predictors, the EPV ratio was approximately 13:1, meeting the widely accepted minimum sample-size requirement for prediction-model development. VIF analysis confirmed no substantial multicollinearity among the seven predictors, with all VIF values below 2.5 (maximum 2.45) (Figure S1).

### Machine learning model performance

The LightGBM model achieved the highest bootstrap-validated AUC of 0.799 (95% CI: 0.699–0.899), followed by LR (0.781), XGBoost (0.776) and RF (0.759). LightGBM also yielded the highest PR-AUC (0.574) and the lowest Brier score (0.151) (Figure 3A–C).

**Figure 3.**
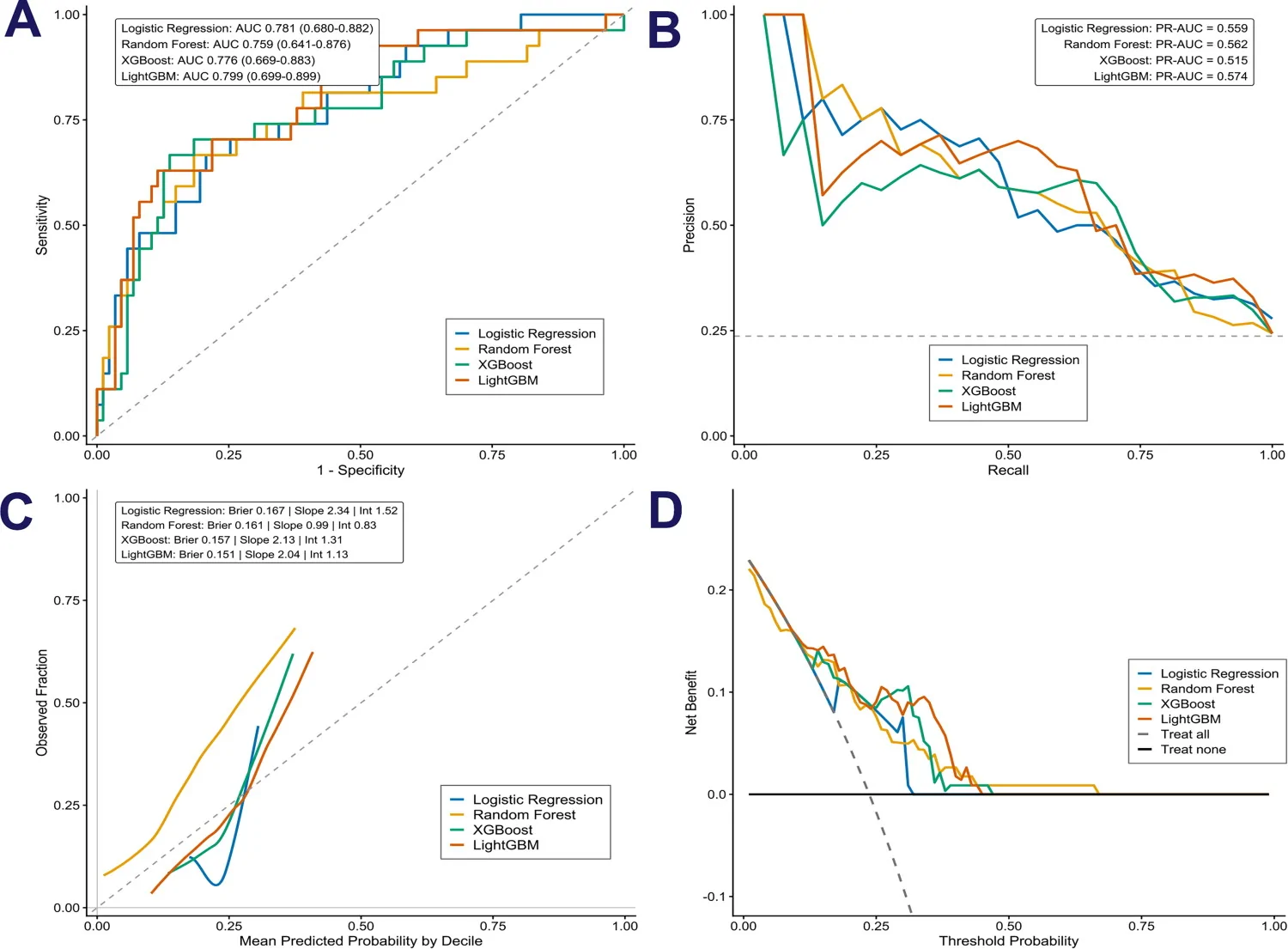
Performance comparison of LR, RF, XGBoost, and LightGBM models for predicting post-PCI NR. (A) ROC curves. LightGBM achieved the highest AUC (0.799, 95% CI: 0.699–0.899), followed by LR (0.781), XGBoost (0.776), and RF (0.759). (B) Precision-recall curves. LightGBM yielded the highest PR-AUC (0.574). (C) Calibration curves. LightGBM had the lowest Brier score (0.151). (D) Decision curve analysis. All four models yielded positive net clinical benefit at threshold probabilities of 0–0.3; within 0.3–0.5, only LightGBM retained modest positive net benefit.

DCA demonstrated that all four models yielded positive net clinical benefit compared with treat-all and treat-none strategies for threshold probabilities ranging from 0 to 0.3. At thresholds of 0.3–0.5, only LightGBM maintained modest positive net clinical benefit, whereas the net benefit of the other three models diminished substantially. Across nine pre-specified performance metrics (AUC, PR-AUC, Brier score, accuracy, precision, sensitivity, specificity, F1, G-mean), LightGBM achieved the best average rank and was selected as the final optimal model (Figure 3D, Table 2).

**Table 2.** Performance metrics of four predictive models for post-PCI NR.

Model	Accuracy	Precision	Sensitivity	Specificity	F1	G-mean	Brier	Cal_Intercept	Cal_Slope
LR	0.763	0.500	0.667	0.793	0.571	0.727	0.167	1.518	2.340
RF	0.781	0.529	0.667	0.816	0.590	0.738	0.161	0.829	0.992
XGBoost	0.816	0.600	0.667	0.862	0.632	0.758	0.157	1.315	2.131
LightGBM	0.825	0.630	0.630	0.885	0.630	0.746	0.151	1.125	2.037

*Note:* Performance metrics of the four predictive models for post-PCI NR. LR, logistic regression; RF, random forest; XGBoost, extreme gradient boosting; LightGBM, Light Gradient Boosting Machine; G-mean, geometric mean; Cal_Intercept, calibration intercept; Cal_Slope, calibration slope.

### SHAP interpretation and risk threshold

SHAP-based model interpretation identified AMI subtype as the strongest contributor to model output (mean absolute SHAP value = 0.581), with neutrophil count ranking second overall (0.236). The inflammatory composite indices NLR (0.151) and MHR (0.138) showed meaningful predictive importance, comparable to albumin (0.156). By contrast, stent number (0.120) and hypertension (0.099) made relatively modest contributions to model outputs. Elevated neutrophil count, NLR, and MHR were consistently linked to positive SHAP values, indicating higher NR risk. STEMI status was associated with increased NR risk, whereas NSTEMI was associated with lower risk (Figure 4A and B).

**Figure 4.**
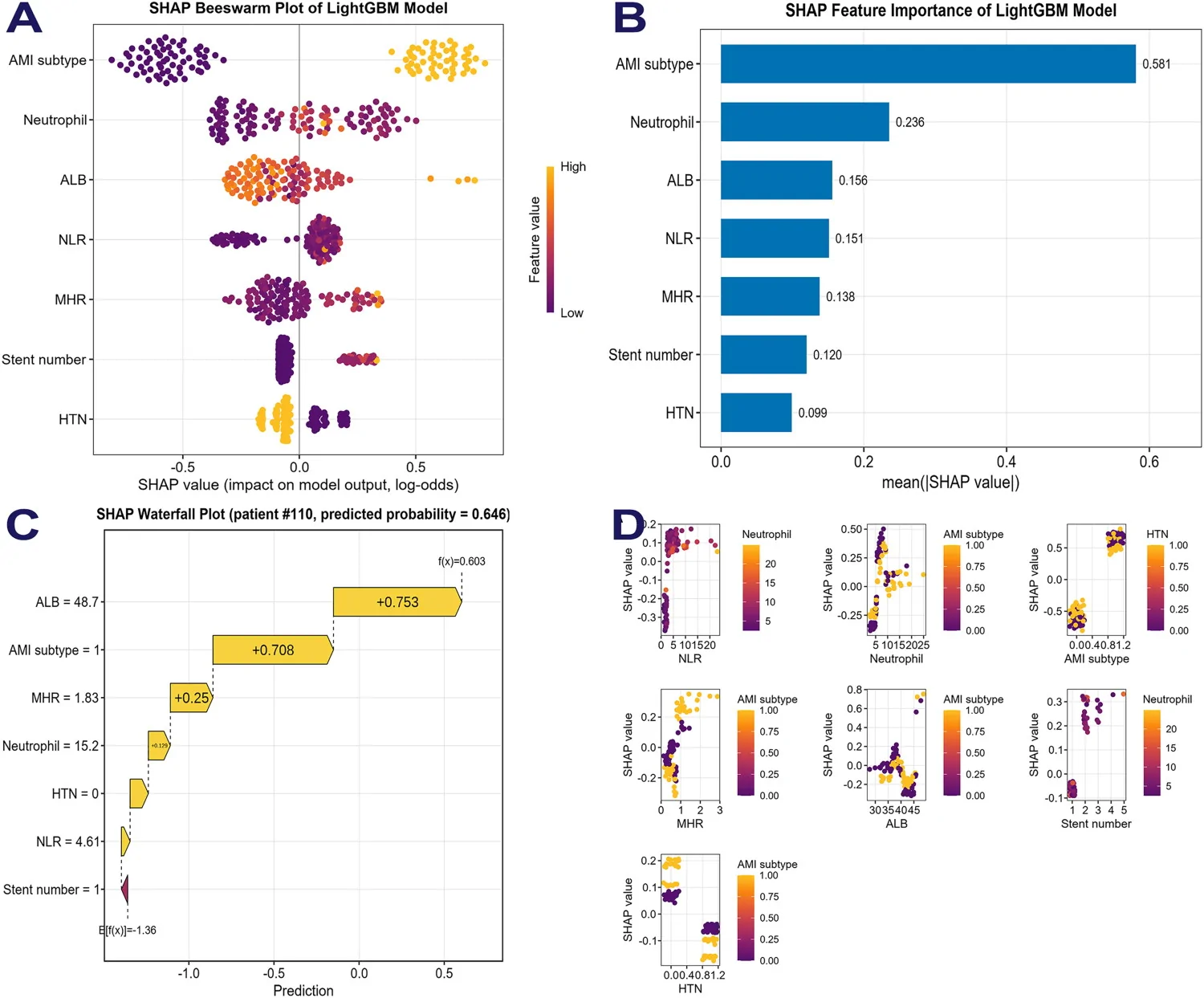
SHAP interpretation of the optimal LightGBM-based model for post-PCI NR prediction. (A) SHAP beeswarm plot. Each dot represents an individual patient. The x-axis corresponds to SHAP values reflecting feature-driven impact on model log-odds output; the colour gradient encodes feature values (purple = low, yellow = high). (B) SHAP-derived feature-importance bar plot, with predictors ranked according to mean absolute SHAP values. (C) SHAP waterfall plot demonstrating feature-level contributions to the predicted log-odds of NR for a representative patient (#110; predicted probability = 0.646). (D) SHAP dependence plots illustrating the relationship between each predictor’s feature value and the corresponding SHAP value for the seven selected variables.

SHAP dependence plots illustrated the relationships between these inflammatory biomarkers and other covariates with model predictions (Figure 4D). Consistent with RCS findings, ALB showed a nonlinear pattern: intermediate levels corresponded to negative SHAP values (lower NR risk), while both low and high ALB levels produced positive SHAP values (higher NR risk). NLR and MHR exhibited positive dose‑response patterns, with higher values of these inflammatory indices translating to stronger risk‑promoting effects. The SHAP waterfall plot for a representative patient (Patient #110, predicted probability = 0.646; log-odds output f(*x*) = 0.603) further underscored the substantial contributions of albumin and inflammatory biomarkers to NR risk estimation (Figure 4C). Of note, SHAP values quantify the contribution of each predictor to the fitted model output and should be interpreted as model-based feature attributions rather than causal effects or fully independent biological contributions, particularly for correlated inflammatory indices.

Beyond SHAP-derived feature interpretation, we defined a risk-stratification cut-off using predicted probabilities generated from the full‑cohort LightGBM model. The Youden-optimal cut-off for high-risk stratification was 0.334. Derived from the same dataset used for model development, this threshold is cohort-specific. Its performance in independent external populations remains unknown, and recalibration will be required prior to real-world clinical application. Patients with a model-predicted NR probability ≥0.334 were categorised as high‑risk in the present study.

### Incremental predictive value of inflammatory markers for post-PCI NR

The baseline clinical model comprised the four LASSO-retained non-inflammatory predictors, namely albumin, hypertension, stent number and AMI subtype. Albumin was included in the baseline model as a nutritional and clinical marker, not as an inflammatory index, to enable separate assessment of the incremental value of each inflammatory biomarker. The 15 composite inflammatory indices together with neutrophil count were each added in turn (Table 3). Neut produced the largest gain in discrimination (ΔAUC +0.029, *p* = 0.024), and NAR, NHR, SII and PIV also significantly improved the AUC, whereas ΔAUC changes for the remaining markers were non-significant. IDI improved significantly for most model extensions, the only exceptions being PHR, UAR, PLR, NMR, LHR and LAR; Neut and SIRI showed the largest IDI (+0.036 each). Significant continuous NRI was observed for NAR, Neut, NHR, SIRI, SII, MHR and PIV, indicating meaningful patient-level risk reclassification. Beyond Neut, which improved both discrimination and reclassification, NAR, NHR, SII and PIV also improved both metrics, whereas NLR and MHR—although retained by LASSO—mainly contributed through reclassification/IDI gains (NLR: IDI *p* = 0.002; MHR: NRI *p* = 0.010, IDI *p* < 0.001).

**Table 3.** Incremental predictive value of the baseline clinical model after addition of different inflammatory markers for post-PCI NR in patients with AMI.

Model	ΔAUC	*P-*ΔAUC	NRI	NRI 95% CI	*P*-NRI	IDI	IDI 95% CI	*P*-IDI
Baseline + Neut	0.029	0.024*	0.465	0.208 – 0.715	<0.001*	0.036	0.008 – 0.082	<0.001*
Baseline + NAR	0.027	0.031*	0.472	0.193 – 0.696	0.002*	0.032	0.006 – 0.077	0.002*
Baseline + NHR	0.026	0.033*	0.430	0.166 – 0.682	0.002*	0.035	0.006 – 0.085	<0.001*
Baseline + SII	0.022	0.047*	0.332	0.091 – 0.602	0.010*	0.024	0.003 – 0.061	0.004*
Baseline + SIRI	0.021	0.069	0.335	0.066 – 0.545	0.014*	0.036	0.007 – 0.078	<0.001*
Baseline + PIV	0.020	0.043*	0.244	0.025 – 0.601	0.038*	0.025	0.004 – 0.069	<0.001*
Baseline + NLR	0.018	0.122	0.215	−0.017 – 0.464	0.066	0.024	0.003 – 0.061	0.002*
Baseline + CAR	0.017	0.132	0.127	−0.115 – 0.379	0.336	0.018	0.001 – 0.060	0.014*
Baseline + MHR	0.015	0.241	0.270	0.066 – 0.553	0.010*	0.032	0.006 – 0.079	<0.001*
Baseline + PNR	0.012	0.297	0.215	−0.009 – 0.498	0.062	0.016	0.001 – 0.049	0.010*
Baseline + UAR	0.007	0.419	0.182	−0.066 – 0.449	0.150	0.006	−0.001 – 0.036	0.082
Baseline + PHR	0.006	0.501	0.274	−0.052 – 0.495	0.098	0.010	−0.001 – 0.040	0.074
Baseline + PLR	0.006	0.457	0.120	−0.087 – 0.366	0.292	0.006	−0.001 – 0.032	0.068
Baseline + NMR	0.003	0.640	0.093	−0.146 – 0.373	0.342	0.004	−0.001 – 0.026	0.088
Baseline + LHR	0.001	0.599	0.007	−0.226 – 0.346	0.796	0.001	−0.001 – 0.009	0.096
Baseline + LAR	0.001	0.958	−0.023	−0.311 – 0.335	0.848	0.001	−0.001 – 0.011	0.158

*Note:* The baseline model includes albumin, hypertension, stent number and AMI subtype. ΔAUC = increment in receiver-operating-characteristic-area-under-curve; 95% CIs are provided for continuous-NRI and IDI. AUCs of nested models were compared using the DeLong test. Continuous-NRI = continuous net reclassification improvement; IDI = integrated discrimination improvement. All incremental-value analyses are exploratory without multiple-comparison adjustment. *p < 0.05.

### Subgroup analysis by AMI subtype

Of 382 patients, 187 (49.0%) had STEMI and 195 (51.0%) had NSTEMI. The incidence of NR was markedly higher in STEMI (38.0%, 71/187) than in NSTEMI (10.3%, 20/195). Stratified regression analyses showed that MHR (OR = 2.860, 95% CI: 1.510–5.830, *p* = 0.002), Neut (OR = 1.130, 95% CI: 1.050–1.230, *p* = 0.001) and NLR (OR = 1.140, 95% CI: 1.040–1.270, *p* = 0.006) were significantly associated with NR in the STEMI subgroup. For ALB, the association did not reach statistical significance (OR = 1.080, 95% CI: 1.000–1.160, *p* = 0.051), whereas hypertension and stent number showed no significant associations. No variables reached statistical significance in the NSTEMI subgroup (all *p* > 0.050). Six interaction tests assessing effect modification by AMI subtype yielded non-significant *P*-interaction values for all six variables (ALB = 0.184, HTN = 0.509, MHR = 0.214, Neut = 0.607, NLR = 0.122, Stent Num = 0.941), none of which met the Bonferroni-corrected significance threshold of *α* = 0.0083 (Figure S2).

## Discussion

NR remains a frequent, prognostically important complication of primary PCI in AMI, reflecting inflammatory microvascular injury rather than mechanical obstruction alone [15,16]. We systematically compared 15 composite immune-inflammatory indices and the neutrophil count for NR prediction, quantified their independent and incremental value over clinical variables, and embedded the informative markers in an interpretable machine learning model. Three findings merit emphasis: a coherent set of neutrophil-centred biomarkers was independently associated with NR; the neutrophil count in particular improved discrimination and reclassification beyond clinical variables; and albumin showed a *U*-shaped, nonlinear relationship with NR that linear models cannot capture.

Reperfusion triggers an acute, neutrophil-centred response: activated neutrophils accumulate in the coronary microcirculation, injure endothelial cells, release proteases and reactive oxygen species, and extrude neutrophil extracellular traps that scaffold platelet adhesion and expose tissue factor, coupling innate immunity to local thrombosis [17–20]. This framework explains why an elevated admission neutrophil count independently predicted NR and why several derived composites (SII, SIRI, NLR, NHR, NAR and PIV) remained predictive after full adjustment. The consistency of the signal across mathematically distinct formulations, in line with earlier STEMI reports [21], argues that neutrophil activation is a shared upstream driver of NR rather than a statistical artefact.

Inflammatory biomarkers carried predictive information beyond conventional clinical variables. With a baseline model comprising albumin, hypertension, stent number and AMI subtype, adding the neutrophil count improved discrimination (ΔAUC +0.029, *p* = 0.024) and, more evidently, reclassification and integrated discrimination (continuous NRI 0.465, *p* < 0.001; IDI +0.036, *p* < 0.001). NAR, NHR, SII and PIV also improved the AUC and, together with SIRI, yielded concordant NRI/IDI gains, whereas PHR, UAR, PLR, NMR, LHR and LAR added no reliable information. Because ΔAUC is conservative once a strong predictor such as AMI subtype is already in the baseline model, the concordant NRI and IDI improvements provide complementary evidence of genuine incremental utility. Moreover, as these markers are derived from routine blood counts at no additional cost, this gain is directly translatable into practice.

RCS analysis identified clinically meaningful nonlinear associations between inflammatory biomarkers and post-PCI NR, with a distinctive *U*-shaped relationship observed for albumin. Albumin is a pleiotropic circulating protein with antioxidant, anti-inflammatory, antithrombotic, and endothelial-protective properties [22]. Lower albumin levels may reflect inflammatory consumption and depleted nutritional reserve [23,24]. By contrast, higher albumin concentrations observed in this subset of patients with early AMI may result from acute volume shifts and haemoconcentration rather than improved nutritional status [25,26]. This non-monotonic *U*-shaped association explains why albumin was retained in the LASSO-selected predictor set and exhibited substantial SHAP importance despite its complex nonlinear relationship with NR risk. SII and PIV demonstrated threshold-like rising curves, indicating that microvascular injury risk increases disproportionately once inflammatory burden exceeds a critical threshold.

Not every index carried stable information. NAR attained a large adjusted odds ratio (OR = 51.947, 95% CI: 3.860–699.039) but was eliminated by LASSO, indicating unstable estimation driven by inter-individual variability and overlap with its component variables; NLR and MHR, by contrast, were estimated stably and retained. Our data do not contradict earlier reports of a neutrophil percentage-to-albumin ratio predicting intraprocedural NR [27]. Instead, they illustrate that, when highly correlated indices compete within one model in our dataset, parsimonious and numerically stable markers tend to be retained. Direct comparison incorporating collinearity diagnostics and regularised selection is therefore preferable to endorsing any single marker, and may partly explain the heterogeneity of earlier single-marker studies.

Among the four tested algorithms, LightGBM achieved the optimal overall performance, with a bootstrap-validated AUC of 0.799, the lowest Brier score, the highest PR-AUC, and the best average rank. SHAP analysis identified AMI subtype and neutrophil count as the top predictive contributors, followed by albumin, NLR and MHR. Consistent effect directions across SHAP, regression and RCS analyses support the biological plausibility of these associations *via* internal triangulation. This model serves as a peri-procedural risk-stratification aid rather than a definitive decision rule. Using the cohort-derived threshold of 0.334, clinicians may identify high-risk patients for enhanced monitoring, though final management should incorporate intraprocedural findings. For patients identified as high‑risk, operators may consider a more cautious procedural strategy, including gentle wire and balloon handling, avoidance of unnecessary high-pressure balloon dilation, earlier consideration of intracoronary vasodilators (e.g. verapamil, nicorandil, and adenosine), and closer haemodynamic and electrocardiographic monitoring; these suggestions are hypothesis-generating and remain to be validated in prospective interventional studies. Of note, despite its favourable discrimination, LightGBM showed systematic miscalibration (calibration intercept = 1.125, slope = 2.037). Such bias may emerge in tree-based models when Bayesian hyperparameter optimisation prioritises out-of-fold classification performance over calibration; limited sample size and outcome imbalance may also contribute. External validation and recalibration are therefore required before broader clinical application.

Subgroup analyses suggested a stronger inflammatory signal in STEMI, consistent with previous studies [28,29]: MHR (OR = 2.86, 95% CI: 1.51–5.83), neutrophil count (OR = 1.13, 95% CI: 1.05–1.23) and NLR (OR = 1.14, 95% CI: 1.04–1.27) were significantly associated with NR. Albumin showed a trend towards significance but did not reach the threshold (OR = 1.08, 95% CI: 1.00–1.16, *p* = 0.051), and no variable reached significance in NSTEMI, where NR occurred less frequently (10.3% vs 38.0%). Interaction tests were non-significant for all six variables (*P*‑interaction 0.122–0.941); with only 20 NR events in the NSTEMI subgroup, this null result most likely reflects limited statistical power rather than truly uniform effects across AMI subtypes. Given this small event number, we did not construct separate subtype-specific machine-learning prediction models, as further data splitting would yield subgroups too small to support stable model development. Our multi-layer analytical strategy, comprising head-to-head comparison, nonlinear modelling, regularised selection, bootstrap validation, incremental testing and SHAP interpretation, indicates that the neutrophil-centred association is not an artefact of any single analytical approach.

## Research limitations

Several study limitations merit acknowledgement. First, this single-centre prospective cohort has a moderate sample size. The model was validated only *via* bootstrap internal validation, lacking external validation in independent cohorts, which restricts generalisability; large multicentre, multi-ethnic cohorts are needed for further confirmation. Second, key intraprocedural variables including thrombus burden and aspiration thrombectomy were not collected in the case-report form and could not be analysed. Other intraprocedural variables with substantial missing data were also excluded, introducing potential unmeasured confounding from lesion complexity. Additionally, only pre-procedural biomarkers were assessed, so dynamic biomarker trajectories could not be evaluated. Third, given its observational design, this study can only establish associations instead of causal relationships, though consistent analytical findings and supporting mechanistic literature lend biological plausibility to our results. Finally, the broad confidence interval for NAR underscores cautious interpretation of ratio-derived inflammatory markers in moderately-sized datasets.

## Conclusion

Among AMI patients undergoing PCI, pre-procedural neutrophil-centred inflammatory biomarkers are independently associated with post-PCI NR and yield heterogeneous incremental discrimination and reclassification over standard clinical variables. Albumin exhibits a U-shaped association with NR, with elevated risk at both low and extremely high concentrations. Our interpretable LightGBM model integrating inflammatory, nutritional and clinical predictors achieved favourable discrimination (AUC = 0.799) and net clinical benefit, though recalibration is required prior to clinical application. Using this cohort-specific threshold of 0.334, the model allows peri-procedural identification of high-risk patients for tailored care. No significant interaction was detected between predictors and AMI subtype, despite stronger associations observed in the STEMI subgroup. These findings support low-cost pre-procedural inflammatory biomarker profiling for NR risk stratification, while external multicentre validation remains necessary.

## Supplementary Material

Table S1.docx

Table S2.docx

Appendix.docx

## Data Availability

The datasets generated and analysed in this study are not publicly available owing to patient confidentiality and ethical constraints, but can be obtained from the corresponding authors (Tao Jing and Xinliang Zou) upon reasonable request and with permission of the Ethics Committee of Southwest Hospital, Army Medical University.

## References

[1] Reffelmann T, Kloner RA. The “no-reflow” phenomenon: basic science and clinical correlates. Heart. 2002;87(2):162–168. doi: 10.1136/heart.87.2.162. PMC1767011 11796561

[2] Kim MC, Cho JY, Jeong HC, et al. Long-term clinical outcomes of transient and persistent no reflow phenomena following percutaneous coronary intervention in patients with acute myocardial infarction. Korean Circ J. 2016;46(4):490–498. doi: 10.4070/kcj.2016.46.4.490. PMC4965427 27482257

[3] Ndrepepa G, Tiroch K, Fusaro M, et al. 5-year prognostic value of no-reflow phenomenon after percutaneous coronary intervention in patients with acute myocardial infarction. J Am Coll Cardiol. 2010;55(21):2383–2389. doi: 10.1016/j.jacc.2009.12.054. 20488311

[4] Ndrepepa G, Cassese S, Xhepa E, et al. Coronary no-reflow and adverse events in patients with acute myocardial infarction after percutaneous coronary intervention with current drug-eluting stents and third-generation P2Y(12) inhibitors. Clin Res Cardiol. 2024;113(7):1006–1016. doi: 10.1007/s00392-023-02340-y. PMC11219448 37962571

[5] Howes S, Altaee O, Ramirez A, et al. Breaking the barrier: unraveling the no-reflow phenomenon in cardiovascular medicine. Catheter Cardiovasc Interv. 2025;106(3):1937–1945. doi: 10.1002/ccd.70017. PMC12412354 40692417

[6] Rostami A, Tajlil A, Separham A, et al. Association between neutrophil-to-lymphocyte ratio and the systemic inflammatory immunologic index and the angiographic SYNTAX score and the TIMI flow grade in acute STEMI: a cohort study. J Tehran Heart Cent. 2021;16(4):147–155. doi: 10.18502/jthc.v16i4.8600. PMC9308885 35935551

[7] Esenboğa K, Kurtul A, Yamantürk YY, et al. Systemic immune-inflammation index predicts no-reflow phenomenon after primary percutaneous coronary intervention. Acta Cardiol. 2022;77(1):59–65. doi: 10.1080/00015385.2021.1884786. 33612077

[8] Yu L, Chen J, Zhang J. Meta-analysis of the correlation between inflammatory response indices and no-reflow after PCI in patients with acute STEMI. Am J Transl Res. 2024;16(10):5168–5181. doi: 10.62347/SUQT4991. PMC11558390 39544771

[9] Deng L, Zhao X, Su X, et al. Machine learning to predict no reflow and in-hospital mortality in patients with ST-segment elevation myocardial infarction that underwent primary percutaneous coronary intervention. BMC Med Inform Decis Mak. 2022;22(1):109. doi: 10.1186/s12911-022-01853-2. PMC9036765 35462531

[10] Byrne RA, Rossello X, Coughlan JJ, et al. 2023 ESC Guidelines for the management of acute coronary syndromes. Eur Heart J. 2023;44(38):3720–3826. doi: 10.1093/eurheartj/ehad191. 37622654

[11] Dai C, Liu M, Zhou Y, et al. A score system to predict no-reflow in primary percutaneous coronary intervention: the PIANO Score. Eur J Clin Invest. 2022;52(2):e13686. doi: 10.1111/eci.13686. 34596236

[12] Ajmal M, Chatterjee A, Acharya D. Persistent or recurrent angina following percutaneous coronary revascularization. Curr Cardiol Rep. 2022;24(12):1837–1848. doi: 10.1007/s11886-022-01820-3. 36287295

[13] Brugaletta S, Ortega-Paz L, Gomez-Lara J, et al. Slow flow and no reflow after percutaneous coronary intervention. EuroIntervention. 2026;22(9):e473–88–e488. doi: 10.4244/EIJ-D-25-01346. PMC13123725 42077032

[14] Jeyaprakash P, Mikhail P, Ford TJ, et al. Index of microcirculatory resistance to predict microvascular obstruction in STEMI: a systematic review and meta-analysis. Catheter Cardiovasc Interv. 2024;103(2):249–259. doi: 10.1002/ccd.30943. 38179600

[15] Konijnenberg LSF, Damman P, Duncker DJ, et al. Pathophysiology and diagnosis of coronary microvascular dysfunction in ST-elevation myocardial infarction. Cardiovasc Res. 2020;116(4):787–805. doi: 10.1093/cvr/cvz301. PMC7061278 31710673

[16] Ndrepepa G, Kastrati A. Coronary no-reflow after primary percutaneous coronary intervention-current knowledge on pathophysiology, diagnosis, clinical impact and therapy. J Clin Med. 2023;12(17):5592. doi: 10.3390/jcm12175592. PMC10488607 37685660

[17] Annibali G, Scrocca I, Aranzulla TC, et al. “No-reflow” phenomenon: a contemporary review. J Clin Med. 2022;11(8):2233. doi: 10.3390/jcm11082233. PMC9028464 35456326

[18] Ma Y. Role of neutrophils in cardiac injury and repair following myocardial infarction. Cells. 2021;10(7):1676. doi: 10.3390/cells10071676. PMC8305164 34359844

[19] El Kazzi M, Rayner BS, Chami B, et al. Neutrophil-mediated cardiac damage after acute myocardial infarction: significance of defining a new target cell type for developing cardioprotective drugs. Antioxid Redox Signal. 2020;33(10):689–712. doi: 10.1089/ars.2019.7928. PMC7475094 32517486

[20] Hally KE, Parker OM, Brunton-O’Sullivan MM, et al. Linking neutrophil extracellular traps and platelet activation: a composite biomarker score for predicting outcomes after acute myocardial infarction. Thromb Haemost. 2021;121(12):1637–1649. doi: 10.1055/s-0041-1728763. 33984869

[21] Stakos DA, Kambas K, Konstantinidis T, et al. Expression of functional tissue factor by neutrophil extracellular traps in culprit artery of acute myocardial infarction. Eur Heart J. 2015;36(22):1405–1414. doi: 10.1093/eurheartj/ehv007. PMC4458286 25660055

[22] Manolis AA, Manolis TA, Melita H, et al. Low serum albumin: a neglected predictor in patients with cardiovascular disease. Eur J Intern Med. 2022;102:24–39. doi: 10.1016/j.ejim.2022.05.004. 35537999

[23] Pan D, Chen H. Relationship between serum albumin level and hospitalization duration following percutaneous coronary intervention for acute coronary syndrome. Sci Rep. 2024;14(1):23883. doi: 10.1038/s41598-024-74955-8. PMC11470946 39396090

[24] Kurtul A, Ocek AH, Murat SN, et al. Serum albumin levels on admission are associated with angiographic no-reflow after primary percutaneous coronary intervention in patients with ST-segment elevation myocardial infarction. Angiology. 2015;66(3):278–285. doi: 10.1177/0003319714526035. 24650948

[25] Yang Z-C, Zhang L, Xi Y-B, et al. Association between albumin changes and prognosis in older patients with acute myocardial infarction. Front Med (Lausanne). 2024;11:1508868. doi: 10.3389/fmed.2024.1508868. PMC11788366 39902029

[26] Hayıroğlu Mİ, Altay S. Overlap between nutritional indices in patients with acute coronary syndrome: a focus on albumin. Balkan Med J. 2024;41(5):324–325. doi: 10.4274/balkanmedj.galenos.2024.190724. PMC11588916 39086257

[27] Yavcin O, Yilmaz Y. Neutrophil percentage/albumin ratio as an independent predictor of the no-reflow phenomenon in patients with st-elevation myocardial infarction undergoing primary percutaneous coronary intervention. Diagnostics (Basel). 2025;15(20):2609. doi: 10.3390/diagnostics15202609. PMC12563376 41153281

[28] Babes EE, Radu A-F, Cretu NA, et al. Risk stratification in acute coronary syndromes: the systemic immune-inflammation index as prognostic marker. Med Sci (Basel). 2025;13(3):116. doi: 10.3390/medsci13030116. PMC12371994 40843738

[29] Liu C, Yang F, Hu Y, et al. The relationships between inflammatory biomarkers, plaque characteristics, and macrophage clusters in coronary plaque: a quantitative assessment of macrophages based on optical coherence tomography. Front Cardiovasc Med. 2025;volume12:1625239. 2025. doi: 10.3389/fcvm.2025.1625239. PMC12238026 40636823

