# Supplementary material for: Interpretable machine learning analysis of immune-inflammatory biomarkers for predicting no-reflow in patients with acute myocardial infarction after percutaneous coronary intervention
Source: Ann Med. 2026 Oct 16;58(1):2747562. doi: 10.1080/07853890.2026.2747562 (PMC13678877; doi:10.1080/07853890.2026.2747562)
Supplement: Supplementary file 1 [file IANN_A_2747562_SM3036.docx]

**Appendix.** TRIPOD-AI Checklist for Prediction Model Development

| **Section / Item** | **No.** | **Checklist item** | **Reported in** | **Location** |
| --- | --- | --- | --- | --- |
| Title | 1 | Identify the study as developing a multivariable prediction model, including machine learning (AI) model, in the title | Yes | Title |
| Abstract | 2 | Provide a structured summary of objectives, study design, setting, participants, sample size, predictors, outcome, statistical analysis, results, and conclusions | Yes | Abstract Objective–Conclusion |
| Introduction | 3 | Explain the medical context and rationale for developing the prediction model, including whether the model is for diagnosis or prognosis | Yes | Introduction |
| Introduction | 4 | Specify the study objectives, including whether the study describes the development of the model or validates an existing model | Yes | Abstract Objective; Introduction |
| Methods – Data source | 5 | Describe the study design, data source and dates of recruitment; describe how participants were identified | Yes | Methods 1.1 |
| Methods – Participants | 6 | Specify eligibility criteria, and the sources and methods of selection of participants; describe the flow of participants (screened/excluded/included) | Yes | Methods 1.1; Figure 1 |
| Methods – Outcome | 7 | Clearly define the outcome that is predicted by the model, including how and when assessed | Yes | Methods 1.2 |
| Methods – Predictors | 8 | Define all predictors, including how and when they were measured, and the timing relative to the outcome | Yes | Methods 1.3–1.4 |
| Methods – Sample size | 9 | Report the number of participants and outcome events; report how the sample size was determined (events-per-variable) | Yes | Methods 1.6; Results 2.4 |
| Methods – Missing data | 10 | Describe how missing data were handled (exclusion threshold; multiple imputation details) | Yes | Methods 1.5 |
| Methods – Data preprocessing | 11 | Describe any data transformations or normalisation applied to predictors before modelling | Yes | Methods 1.4, 1.6; Table S1 |
| Methods – Model development | 12 | Specify the type of model, the modelling technique, and the software/packages used | Yes | Methods 1.5–1.6 |
| Methods – Model specification | 13 | Provide the full specification of the final model (predictors, hyperparameters, decision threshold) | Yes | Methods 1.6–1.7; Results 2.4, 2.6; Table S1 |
| Methods – Training | 14 | Describe how the data were used for model development (no train/test split; stratified 5-fold cross-validation for tuning; full cohort for final model) | Yes | Methods 1.6 |
| Methods – Performance | 15 | Specify the primary performance measure and how it was computed (AUC, PR-AUC, Brier, calibration metrics, DCA) | Yes | Methods 1.7 |
| Methods – Model updating | 16 | If the model was updated, describe the updating procedure | N/A | — |
| Methods – Heterogeneity | 17 | If performed, describe subgroup analyses and pre-specified heterogeneity/interaction assessments | Yes | Methods 1.9; Results 2.8 |
| Methods – Development vs evaluation | 18 | Clearly distinguish between model development and evaluation datasets | Yes | Methods 1.6–1.7 |
| Methods – Confidence intervals | 19 | Describe how uncertainty in performance estimates was quantified (2.5th–97.5th percentiles of 1000 bootstrap resamples) | Yes | Methods 1.7 |
| Methods – Fairness | 20 | If applicable, describe assessments of model performance across subgroups to evaluate fairness | Partially | Subgroup analyses by AMI subtype (Methods 1.9; Results 2.8); no formal assessment by sex, age or ethnicity |
| Methods – Validation | 21 | Describe the validation method (internal: 1000-iteration bootstrap); state absence of external validation explicitly | Yes | Methods 1.7; Limitations |
| Methods – Interpretability | 22 | Describe methods used to aid model interpretability (SHAP analyses; Youden-optimal threshold) | Yes | Methods 1.7; Results 2.6 |
| Methods – Existing models | 23 | If comparisons with existing models were made, describe the comparison methods | N/A | — |
| Methods – Predictions | 24 | Describe how the final model predictions are used (probability threshold 0.334 for high-risk stratification) | Yes | Results 2.6; Discussion |
| Methods – Ethics | 25 | Report ethics approval and consent procedures | Yes | Methods 1.1 |
| Methods – Openness | 26 | State whether code and/or data are available and how they can be accessed | Yes | Data Availability Statement |
| Methods – Funding/COI | 27 | Report funding sources, conflicts of interest and acknowledgements | Yes | Conflict of Interest; Funding |
| Results | — | Report participant flow, model performance with confidence intervals, calibration and clinical utility results | Yes | Results 2.1–2.8 |
| Discussion | — | Interpret results, discuss limitations (external validation, missing procedural variables, observational design), and clinical implications | Yes | Discussion |
| Conclusion | — | Summarise findings and the need for external validation | Yes | Conclusion |
