# Supplementary material for: Interpretable machine learning analysis of immune-inflammatory biomarkers for predicting no-reflow in patients with acute myocardial infarction after percutaneous coronary intervention
Source: Ann Med. 2026 Oct 16;58(1):2747562. doi: 10.1080/07853890.2026.2747562 (PMC13678877; doi:10.1080/07853890.2026.2747562)
Supplement: Supplementary file 2 [file IANN_A_2747562_SM3037.docx]

**Table S2.** Comparison of baseline characteristics between NR and normal-flow groups after PCI

| Characteristic | Normal flow | No reflow | *P*-value |
| --- | --- | --- | --- |
|  | N = 291 | N = 91 |  |
| Age (years) | 62 ± 12 | 62 ± 12 | 0.515 |
| Sex (Male) | 233 (80.1%) | 78 (85.7%) | 0.227 |
| LDL-C (mmol/L) | 2.85 ± 1.05 | 2.77 ± 0.86 | 0.477 |
| UA (μmol/L) | 393 ± 117 | 423 ± 148 | 0.078 |
| ALB (g/L) | 39.9 ± 4.3 | 41.3 ± 4.5 | 0.012* |
| hs-CRP (mg/L) | 6.2 (1.4-22.7) | 9.4 (2.1-44.7) | 0.032* |
| HDL-C (mmol/L) | 1.13 ± 0.32 | 1.09 ± 0.29 | 0.338 |
| Mono (×10⁹/L) | 0.69 ± 0.34 | 0.91 ± 0.50 | <0.001* |
| PLT (×10⁹/L) | 229 ± 80 | 253 ± 88 | 0.024* |
| Neut (×10⁹/L) | 6.6 ± 3.3 | 8.9 ± 4.3 | <0.001* |
| Lymph (×10⁹/L) | 1.8 (1.4-2.2) | 1.8 (1.3-2.5) | 0.330 |
| SII | 694 (456-1108) | 953 (651-1516) | <0.001* |
| SIRI | 1.9 (1.2-3.4) | 3.3 (1.8-5.4) | <0.001* |
| NLR | 3.98 ± 2.60 | 5.34 ± 4.14 | 0.004* |
| PLR | 138 ± 68 | 150 ± 85 | 0.213 |
| PNR | 41 ± 18 | 34 ± 18 | 0.002* |
| NHR | 6.2 ± 3.5 | 8.8 ± 5.6 | <0.001* |
| MHR | 0.66 ± 0.36 | 0.90 ± 0.59 | <0.001* |
| PHR | 216 ± 92 | 252 ± 136 | 0.020* |
| LHR | 1.6 (1.2-2.2) | 1.8 (1.3-2.5) | 0.123 |
| NMR | 10.0 ± 3.8 | 11.0 ± 5.8 | 0.136 |
| CAR | 0.158 (0.033-0.629) | 0.212 (0.048-1.135) | 0.047* |
| UAR | 10.0 ± 3.2 | 10.3 ± 3.6 | 0.418 |
| LAR | 0.043 (0.035-0.057) | 0.047 (0.033-0.060) | 0.502 |
| NAR | 0.17 ± 0.09 | 0.22 ± 0.10 | <0.001* |
| PIV | 409 (251-751) | 824 (425-1299) | <0.001* |
| BMI | 24.5 ± 3.5 | 24.4 ± 3.4 | 0.801 |
| SYNTAX | 6.2 ± 4.5 | 7.2 ± 4.8 | 0.097 |
| Stent Num | 1.29 ± 0.61 | 1.36 ± 0.66 | 0.322 |
| Smoking | 190 (65.3%) | 64 (70.3%) | 0.374 |
| HTN | 186 (63.9%) | 46 (50.5%) | 0.023* |
| DM | 115 (39.5%) | 36 (39.6%) | 0.994 |
| AMI subtype (STEMI) | 116 (39.9%) | 71 (78.0%) | <0.001* |
| Pre TIMI |  |  | 0.004* |
| 0 | 96 (33.0%) | 49 (53.8%) |  |
| 1 | 82 (28.2%) | 18 (19.8%) |  |
| 2 | 26 (8.9%) | 4 (4.4%) |  |
| 3 | 87 (29.9%) | 20 (22.0%) |  |

**Note:** Normally-distributed continuous variables are presented as mean ± SD; non-normally-distributed continuous variables as median (interquartile range) [M (Q1, Q3)]. Categorical variables are shown as n (%). An asterisk (*) denotes two-sided *P* < 0.05.
