# Supplementary material for: Interpretable machine learning analysis of immune-inflammatory biomarkers for predicting no-reflow in patients with acute myocardial infarction after percutaneous coronary intervention
Source: Ann Med. 2026 Oct 16;58(1):2747562. doi: 10.1080/07853890.2026.2747562 (PMC13678877; doi:10.1080/07853890.2026.2747562)
Supplement: Supplementary file 3 [file IANN_A_2747562_SM3038.docx]

**Table S1.** Optimised hyperparameters for four predictive models obtained via Bayesian tuning

| Model | Logistic Regression | Random Forest | XGBoost | LightGBM |
| --- | --- | --- | --- | --- |
| Hyperparameters | alpha=1.00000, lambda=0.07757 | mtry=1, nodesize=12, ntree=1000 | eta=0.010, max_depth=6, min_child_weight=4, subsample=1.00, colsample_bytree=0.50, gamma=5.000, nrounds=440 | learning_rate=0.010, num_leaves=31, max_depth=2, feature_fraction=0.50, bagging_fraction=0.50, min_data_in_leaf=36, nrounds=204 |

**Note:** All hyperparameters were optimised via Bayesian optimisation under stratified 5-fold cross-validation on the full cohort (28 evaluations per model; OOF F1-score objective with fold-specific Youden thresholds).
